# Supplementary material for: How maternal morbidities impact women’s quality of life during pregnancy and postpartum in sub-Saharan Africa and South Asia: A qualitative study
Source: PLOS Glob Public Health. 2025 Sep 11;5(9):e0004229. doi: 10.1371/journal.pgph.0004229 (PMC12425202; doi:10.1371/journal.pgph.0004229)
Supplement: S1 File — (PDF) [file pgph.0004229.s001.pdf]

A. Focus Group Discussion Guide: Pregnant Women

Good [morning/afternoon/evening]. My name is [insert], and I will be facilitating the discussion today. This is [insert] and [he/she/they] will be taking notes and assisting with the discussion. Thank you again for taking the time to participate. We will be discussing your experience during pregnancy and are interested in learning about any health and wellbeing challenges or difficulties you or others in your community might have faced related to pregnancy. The information you share with us will be anonymous and treated as confidential. You do not have to answer any questions you do not feel comfortable with and are free to leave the group at any point if you do not want to continue participating in the discussion. The information discussed today will help us to better understand women's experiences during pregnancy and will inform strategies to prevent and better address the health and wellbeing challenges women face during pregnancy to ultimately provide greater support to women during these critical life stages.

Even though we will be taking notes, we are not able to write down everything discussed and would like to be able to go back and listen to any information we might have missed and make sure that we heard you correctly. Is everyone okay with recording the discussion? (confirm that all participants consent)

Okay, great. We will go ahead and start the discussion. We ask that you please take turns while speaking and do not interrupt anyone. We are interested in what all of you have to say and there are no right or wrong answers. The discussion will last about 45 minutes. Does anyone have any questions before we begin?

1. How do women in your community care for their health and wellbeing while pregnant?

    a. How and where do pregnant women seek health care?
    b. What are the reasons women seek health care while pregnant?
    c. How does being pregnant affect the types of activities that women do?
    d. What types of activities are women more likely to do while pregnant?
    e. What types of activities are women less likely to do while pregnant?
    f. How does being pregnant impact women's lifestyle and health behaviors?

2. What are the main health and wellbeing challenges that affect pregnant women?

    a. Can you explain what [insert problem or condition] is?
    b. How common do you think [insert problem or condition] is in your community?
    c. What do you think causes [insert problem or condition]?
    d. How much does [insert problem or condition] interfere with a woman's life or ability to carry out daily tasks and responsibilities?
    e. Can you think of any other challenges or problems pregnant women face?
    f. What are some challenges or problems pregnant women face that may be rare but very severe?

3. How do others in your family or community view the pregnancy-related challenges women face?

a. How do they view physical challenges women face during pregnancy?
b. How do they view emotional challenges women face during pregnancy?
c. How do they view social challenges women face during pregnancy?
d. How do they view economic challenges women face during pregnancy?

4. In your opinion, what are ways that women could be better supported during pregnancy?

5. Is there anything else you'd like to share about women's experiences of pregnancy in your community?

6. Can you think of any other challenges or problems women face during pregnancy that we haven't already discussed?

Okay, that concludes our discussion today. Again, we really appreciate you all taking the time to participate and find the information you shared very valuable.

B. Focus Group Discussion Guide: Late Postpartum Participants (≥6 months)

Good [morning/afternoon/evening]. My name is [insert], and I will be facilitating the discussion today. This is [insert] and [he/she/they] will be taking notes and assisting with the discussion. Thank you again for taking the time to participate. We will be discussing your experiences after childbirth and are interested in learning about any health and wellbeing challenges or difficulties you or others in your community might have faced after childbirth. The information you share with us will be anonymous and treated as confidential. You do not have to answer any questions you do not feel comfortable with and are free to leave the group at any point if you do not want to continue participating in the discussion. The information discussed today will help us to better understand women's postpartum experiences and will inform strategies to prevent and better address the health and wellbeing challenges women face after childbirth to ultimately provide greater support to women during this critical life stage.

Even though we will be taking notes, we are not able to write down everything discussed and would like to be able to go back and listen to any information we might have missed and make sure that we heard you correctly. Is everyone okay with recording the discussion? (confirm that all participants consent)

Okay, great. We will go ahead and start the discussion. We ask that you please take turns while speaking and do not interrupt anyone. We are interested in what all of you have to say and there are no right or wrong answers. The discussion will last about 45 minutes. Does anyone have any questions before we begin?

1. Could you tell me about the birth of your child?

    a. What were your expectations regarding what it would be like to give birth?
        i. Did the birth go as you had planned?
        ii. How difficult or easy was your birth?
        iii. Was there anything you wish had gone differently?

2. How do women in your community care for their health and wellbeing after childbirth?

    a. How and where do women seek health care after childbirth?
    b. What are the reasons women seek health care after childbirth?
    c. How does childbirth affect the types of activities that women typically do?
    d. What types of activities are women more likely to do after childbirth?
    e. What types of activities are women less likely to do after childbirth?

3. What are the main health and wellbeing challenges that affect women after childbirth?

    a. Can you explain what [insert problem or condition] is?
    b. How common do you think [insert problem or condition] is in your community?
    c. What do you think causes [insert problem or condition]?
    d. How much does [insert problem or condition] interfere with a woman's life or ability to do the typical things that women do throughout the day?

        i. [Probe for things like cooking, cleaning, taking care of children, working outside of the home?]

   e. Can you think of any other health and wellbeing challenges or problems women face in the days or weeks after childbirth?
   f. Can you think of any other health and wellbeing challenges or problems women face in the months or year after childbirth?
   g. What are some challenges or problems women face after childbirth that may be rare but very severe?

4. How do others in your family or community view the childbirth-related challenges women face?

   a. How do they view physical challenges women face after childbirth?
   b. How do they view emotional challenges women face after childbirth?
   c. How do they view social challenges women face after childbirth?
   d. How do they view economic challenges women face after childbirth?

5. In your opinion, what are ways that women could be better supported after childbirth?

6. Is there anything else you'd like to share about women's experiences following childbirth in your community?

7. Can you think of any other challenges or problems women face after childbirth that we haven't already discussed?

Okay, that concludes our discussion today. Again, we really appreciate you all taking the time to participate and find the information you shared very valuable.

C. In-Depth Interview Guide: Early Postpartum Participants (≤6 weeks)

Good [morning/afternoon/evening]. My name is [insert], and I will be interviewing you today. Thank you again for taking the time to participate. We are interested in learning about your experience with childbirth and more generally about health and wellbeing challenges women in your community face related to childbirth. The information you share with us will be anonymous and treated as confidential. You do not have to answer any questions I ask you and you are free to stop at any point if you do not want to continue. The information discussed today will help us to better understand women's experiences following childbirth and will inform strategies to prevent and better address the health and wellbeing challenges women face during pregnancy and childbirth to ultimately provide greater support to women during these critical life stages.

I will not be able to write down everything you tell me and would like to be able to go back and listen to any information I might have missed and make sure that I heard you correctly. Are you okay with recording the interview? (confirm that the participant consents)

Okay, great. We will go ahead and start. The interview will last about 45 minutes. Do you have any questions before we begin?

1. Could you tell me about the birth of your child?

    a. What were your expectations regarding what it would be like to give birth?
        i. Did the birth go as you had planned?
        ii. How difficult or easy was your birth?
        iii. Was there anything you wish had gone differently?

    b. What type of support, for example from friends, family, a midwife, or doctor, did you receive during the birth?

    [if participant indicates having complications, thank them for sharing and ask if they would be willing to answer some additional complications about their experience at the end of the interview and then continue with question #2 below]

    [if participant does not indicate having complications, continue with question #2 below]

2. How do women in your community care for their health and wellbeing after childbirth?

    a. How and where do women seek health care after childbirth?
    b. What are the reasons women seek health care after childbirth?
    c. How do you think childbirth affects the types of activities that women typically do?
    d. What types of activities are women more likely to do after childbirth?
    e. What types of activities are women less likely to do after childbirth?

3. What are the main health and wellbeing challenges that you think affect women after childbirth?

    a. Can you explain what [insert problem or condition] is?

b. How common do you think [insert problem or condition] is in your community?
c. What do you think causes [insert problem or condition]?
d. How much does [insert problem or condition] interfere with a woman's life or ability to do typical activities throughout the day?
    i. [Probe for things like cooking, cleaning, taking care of children, working outside of the home?]
e. Can you think of any other health and wellbeing challenges or problems women face in the days or weeks after childbirth?
f. Can you think of any other health and wellbeing challenges or problems women face in the months or year after childbirth?
g. What are some challenges or problems women face after childbirth that may be rare but very severe?

4. How do others in your family or community view the childbirth-related challenges women face?

    a. How do they view physical challenges women face after childbirth?
    b. How do they view emotional challenges women face after childbirth?
    c. How do they view social challenges women face after childbirth?
    d. How do they view economic challenges women face after childbirth?

5. In your opinion, what are ways that women could be better supported after childbirth?

6. Is there anything else you'd like to share about women's experiences following childbirth in your community?

7. Can you think of any other challenges or problems women face after childbirth that we haven't already discussed?

[If the participant indicated having complications, continue to "Extended Interview Questions" below]

[If the participant did not indicate having complications: Okay, that concludes our discussion today. Again, we really appreciate you all taking the time to participate and find the information you shared very valuable.]

Extended Interview Questions (If Participant Indicated Complications)

Now, we will be discussing your personal experience following the birth of your child and are interested in learning about any specific health and wellbeing challenges or difficulties you might have faced related to childbirth. As in the first portion of this interview, the information you share with us will be anonymous and treated as confidential. You do not have to answer any questions I ask you and you are free to stop at any point if you do not want to continue. The information discussed today will help us to better understand women's experiences following childbirth and will inform strategies to prevent and better address the health and wellbeing challenges women face during pregnancy and childbirth to ultimately provide greater support to women during these critical life stages. Do you have any questions before we begin?

1. What complications did you experience **leading up to or during** birth?

    i. Was [insert complication] unexpected?
    ii. Can you tell me more about [insert] complication]?
    iii. How did you find out you had [insert complication]?
    iv. What do you think caused [insert complication]?
    v. What is/was it like to have [insert complication]?
    vi. How long did [insert condition] last/has [insert condition] lasted?
    vii. How does/did having [insert condition] affect your life?
        1. Does/did it affect you physically?
        2. Does/did it affect you mentally?
        3. Does/did it affect you financially?
        4. Does/did it affect how you manage your household (chores)?
        5. Does/did it affect your social life with your friends and neighbors?
        6. Does/did it affect your relationship with your husband/boyfriend/partner/romantic life?
        7. Does/did it affect your relationship with other family members?
    viii. How does/did it make you feel?

2. What health and wellbeing problems or challenges have you encountered **following** the birth of your child? This could be in the days, weeks, or months after childbirth. Can you tell me more about your experience?

    a. How did you find out you had/have [insert condition]?
    b. What do you think caused the [insert condition]?
    c. What is/was it like to have [insert condition]?
    d. Can you describe when the [insert condition] started?
        i. How long did [insert condition] last/has [insert condition] lasted?
        ii. How does/did having [insert condition] affect your life?
        iii. Does/did it affect you physically?
        iv. Does/did it affect you mentally?
        v. Does/did it affect you financially?
        vi. Does/did it affect how you manage your household (chores)?
        vii. Does/did it affect your social life with your friends and neighbors?

viii. Does/did it affect your relationship with your husband/boyfriend/partner/romantic life?
ix. Does/did it affect your relationship with other family members?
x. How does/did it make you feel?
xi. Can you think of any other health and wellbeing problems or challenges you encountered in the days, weeks, or months after childbirth?

3. Is your doctor or midwife aware of these challenges?

    a. What type of support did your doctor or midwife provide for this complication?
    b. Did you notice/have you noticed any changes in the way your doctor or midwife treated you as a woman with [insert condition(s) or challenge(s)]?
    c. Do/did you feel supported by your doctor or midwife in navigating [insert condition(s) or challenge(s)]?
        i. Why or why not?

4. Are your family, friends, or community aware of these challenges?
    a. Did the things you typically do throughout the day change due to these health and wellbeing challenges?
    b. Did your family, friends, or community's expectations around what you would be able to do change?
        i. Why or why not?
    c. Did you notice/have you noticed any changes in the way your family, partner/husband/boyfriend, friends, or community treated you as a woman with [insert condition or challenge]?
    d. Do/did you feel supported by your family, partner/husband/boyfriend, friends, or community in navigating [insert condition or challenge]?
        i. Why or why not?

5. Can you think of any other challenges you faced following the birth of your child we haven't already discussed?

6. Is there anything else you'd like to share about your experience following the birth of your child?

Okay, that concludes the interview today. We really appreciate you taking the time to participate and find the information you shared very valuable.
