# Supplementary material for: How maternal morbidities impact women’s quality of life during pregnancy and postpartum in sub-Saharan Africa and South Asia: A qualitative study
Source: PLOS Glob Public Health. 2025 Sep 11;5(9):e0004229. doi: 10.1371/journal.pgph.0004229 (PMC12425202; doi:10.1371/journal.pgph.0004229)
Supplement: S2 File — (DOCX) [file pgph.0004229.s002.docx]

| **Codes** | **Overarching Theme 1** | **Minor Themes** |
| --- | --- | --- |
| Difficulty with chores and strenuous activities  Emotional challenges  Stress  Breastfeeding challenges  Difficulty with self-care | Physical and emotional symptoms pose a barrier to daily activities | Physical symptoms make chores difficult  Emotional changes adversely impact daily functioning |
| **Codes** | **Overarching Theme 2** | **Minor Themes** |
| Lack of support from community  Gender expectations and discrimination  Lack of medical support and awareness  Family is not supportive  Baby's father is not supportive  Mistreatment by baby's father  Judgement and resentment | Lack of social support detracts from women's HRQoL | Lack of support from the baby's father  Mistreatment from the baby's father  Lack of support from the community  Lack of support from family members  Concern around the sex of the baby |
| **Codes** | **Overarching Theme 3** | **Minor Themes** |
| Baby's father provides non-financial support  Family provides financial support  Family provides non-financial support  Community provides financial support  Community provides non-financial support | Receipt of social support mitigates adverse impacts of pregnancy and postpartum symptoms on HRQoL | Support from family  Support from community  Support from husband |
| **Codes** | **Overarching Theme 4** | **Minor Themes** |
| Nutritional challenges  Financial challenges  Transportation challenges  Unable to continue employment  Lack of childcare  Lack of necessities | Economic challenges exacerbate declines in women’s HRQoL during pregnancy and postpartum | Inability to afford necessities  Employment challenges postpartum |
