# Supplementary material for: How maternal morbidities impact women’s quality of life during pregnancy and postpartum in sub-Saharan Africa and South Asia: A qualitative study
Source: PLOS Glob Public Health. 2025 Sep 11;5(9):e0004229. doi: 10.1371/journal.pgph.0004229 (PMC12425202; doi:10.1371/journal.pgph.0004229)
Supplement: Attachment — Submitted filename: Review_Plos_Global.docx [file pgph.0004229.s006.docx]

**Review**

Congratulations on crafting a well-written and critical manuscript. I enjoyed reviewing your work. This manuscript identifies the physical and emotional challenges faced, factors influencing their quality of life, and lived experiences of maternal morbidities, which is of paramount importance, particularly in LMICs in sub-Sahara, Africa and Southeast Asia. Below are items for review. The items are listed under the subheadings as major and minor points for your consideration.

**Major concerns**

1. Some quotes reveal reports of concerning themes such as violence, psychological/verbal abuse, and societal pressures/judegement/rejection. It may be helpful to ask whether interviewers were trained to provide appropriate support or referrals to participants disclosing abuse or trauma, and whether ethics protocols covered this. If this was done please mention it in the methods section. If not, please add it to the limitations section of the discussion.
2. Abstract line 45: The conclusions read more as the results. For example, the conclusions listed on page 18, lines 391-393, should be adapted for the abstract. Please list the primary outcomes in more detail in the abstract section as results. This can be done by providing examples. How do the results differ or agree between African and Southeast Asian countries?
3. Line 61: what percentage of MMR is those listed for Southeast Asia?
4. Line 67: and for East Asia or Southeast Asia? Please keep comparisons uniform throughout the introduction section.
5. Were translators needed for the direct quotes in the manuscript's results section? Did the study participant answer in English? If not, how were the quotes translated, and how were accuracy and the intended meaning maintained? Please add these methodologies and descriptions to the materials and methods section (Line 122). Please expand what is the intended meaning of “as per standardised translation protocol”? (Line 144) Is there a reference that can be provided here? Line 151: by whom were the transcripts reviewed? Although you mention asking for clarification about translation, the manuscript lacks a direct statement about the *language(s) used* in the interviews and whether any issues arose during translation and back-translation. It’s worth explicitly recommending that this be stated more clearly.
7. To guide the reader, please add and figures to the manuscript's main text.
8. Please expand on the intended meaning of “probe”. The authors may consider rephrasing the sentence to clarify the intended meaning.
9. Please provide a citation for Dedoose.
   a. <https://uk.sagepub.com/en-gb/eur/qualitative-and-mixed-methods-data-analysis-using-dedoose/book258543>
10. Line 184: How does the unemployment statistic compare to the national averages? This may be a more valid point for the discussion section. Are the participants skewed towards unemployment? Are LMICS more likely to be unemployed during pregnancy? Please elaborate. Does this contribute to economic challenges listed as thematic unit 4?
11. Line 333: Please provide more references for the existing literature alluded to. Can you add comparisons to other Sub-Saharan African countries or South East Asian countries?
12. Please add a succinct paragraph in the discussion section on how these challenges can be overcome. For example, can this work be used to advocate for these women's well-being, for postnatal care, food supply, transportation, and medicines in local settings, or serve as a baseline to advocate for increased awareness and education in these communities? What are the next steps following this study?
13. Line 393, please provide references where this work has consistent outcomes to those listed here.
14. Line 396: how can these educational challenges be implemented?
15. In line 90, the authors state, "Findings will inform development of tailored interventions to improve the QoL of childbearing women in LMICs.” Can the authors please expand on these taylord approaches in the discussion section
16. While the manuscript identifies four overarching themes, it doesn’t explicitly compare how experiences differed or were similar between countries or regions. A more nuanced discussion of cross-country comparisons may be required, particularly between Sub-Saharan Africa and South Asia. This can be done succinctly in the results section and in a short section within the discussion section

**Minor concerns**

1. Line 58 “Despite recent global advancements,” please name the recent advancements succinctly.
2. Line 62 and 63. Please add a comma after tip of the iceberg and after severe maternal morbidity
3. Please add a “the” before development
4. Replace global push with global drive
5. The use of AI-derived text often includes the overuse of an em dash (—). This is great to emphasise text, but a good writing tip is to limit it to once per manuscript. Overuse is distracting to the reader. For example, it is used in lines 62, 77, 78, 358 (x2), 370, 371, 388 and 388. Another tip to the authors is to avoid using “underscore” as this is widely used in AI-generated text and can become distracting.
6. In the first sentence of the results section, please remove the word “who”.
7. Please replace a with “an” FGD “participated in a FGD during pregnancy”.
8. In the following sentence, please change morbidities to morbidity: “In some cases, this also manifested as physical morbidities”.
9. Line 344 change “review on” to review “of”.
10. Please change increase to increasing (Line 375)
11. Ensure the consistent use of a space or the percentage sign (such as “75%” vs “75 %”).
12. The abstract does not mention the number of study sites or countries. Pleaser add it.
