# Supplementary material for: How maternal morbidities impact women’s quality of life during pregnancy and postpartum in sub-Saharan Africa and South Asia: A qualitative study
Source: PLOS Glob Public Health. 2025 Sep 11;5(9):e0004229. doi: 10.1371/journal.pgph.0004229 (PMC12425202; doi:10.1371/journal.pgph.0004229)
Supplement: Attachment — Submitted filename: Response-to-Reviewers-26JUN2025.docx [file pgph.0004229.s007.docx]

**Response to Reviewers**

**NOTE: Page and line numbers align with the revised manuscript with tracked changes document.**

1. Please include a complete copy of PLOS’ questionnaire on inclusivity in global research in your revised manuscript. Our policy for research in this area aims to improve transparency in the reporting of research performed outside of researchers’ own country or community. The policy applies to researchers who have travelled to a different country to conduct research, research with Indigenous populations or their lands, and research on cultural artefacts. The questionnaire can also be requested at the journal’s discretion for any other submissions, even if these conditions are not met.  Please find more information on the policy and a link to download a blank copy of the questionnaire here: <https://journals.plos.org/globalpublichealth/s/best-practices-in-research-reporting>. Please upload a completed version of your questionnaire as Supporting Information when you resubmit your manuscript.

**RESPONSE: Thank you. The questionnaire is now included.**

2. We do not publish any copyright or trademark symbols that usually accompany proprietary names, eg (R), (C), or TM  (e.g. next to drug or reagent names). Please remove all instances of trademark/copyright symbols throughout the text, including ™  on page 8.

**RESPONSE: We have removed these symbols as requested.**

3. Tables should not be uploaded as individual files. Please remove these files and include the Tables in your manuscript file as editable, cell-based objects. For more information about how to format tables, see our guidelines:

<https://journals.plos.org/globalpublichealth/s/tables>

**RESPONSE: Tables are now included in the manuscript file.**

3. We have noticed that you have uploaded Supporting Information files, but you have not included a list of legends. Please add a full list of legends for your Supporting Information files after the references list.

**RESPONSE: We have added a list of legends, thank you. Please see page 41.**

Additional Editor Comments (if provided):


[Note: HTML markup is below. Please do not edit.]

Reviewers' comments:

Reviewer's Responses to Questions

Comments to the Author

1. Does this manuscript meet PLOS Global Public Health’s [publication criteria](https://track.editorialmanager.com/CL0/https:%2F%2Fjournals.plos.org%2Fglobalpublichealth%2Fs%2Fcriteria-for-publication/1/010f0196c7d0e728-d9efb81e-d2d9-44d3-b4e8-b9ffc3e8a644-000000/urocHPA6z9UnmW39B1hx9KUcWlz1K63-NZYvJmvDbwk=210)? Is the manuscript technically sound, and do the data support the conclusions? The manuscript must describe methodologically and ethically rigorous research with conclusions that are appropriately drawn based on the data presented.

Reviewer #1: Partly

Reviewer #2: Yes

Reviewer #3: Yes

2. Has the statistical analysis been performed appropriately and rigorously?

Reviewer #1: Yes

Reviewer #2: Yes

Reviewer #3: N/A

3. Have the authors made all data underlying the findings in their manuscript fully available (please refer to the Data Availability Statement at the start of the manuscript PDF file)?

The [PLOS Data policy](https://track.editorialmanager.com/CL0/https:%2F%2Fjournals.plos.org%2Fglobalpublichealth%2Fs%2Fdata-availability/1/010f0196c7d0e728-d9efb81e-d2d9-44d3-b4e8-b9ffc3e8a644-000000/jEj_ZwFwB7aXUA_B6-xWA8Lz2A5HDJoFTNhxACm4xMI=210) requires authors to make all data underlying the findings described in their manuscript fully available without restriction, with rare exception. The data should be provided as part of the manuscript or its supporting information, or deposited to a public repository. For example, in addition to summary statistics, the data points behind means, medians and variance measures should be available. If there are restrictions on publicly sharing data—e.g. participant privacy or use of data from a third party—those must be specified.

Reviewer #1: Yes

Reviewer #2: Yes

Reviewer #3: Yes

4. Is the manuscript presented in an intelligible fashion and written in standard English?

PLOS Global Public Health does not copyedit accepted manuscripts, so the language in submitted articles must be clear, correct, and unambiguous. Any typographical or grammatical errors should be corrected at revision, so please note any specific errors here.

Reviewer #1: Yes

Reviewer #2: Yes

Reviewer #3: Yes

5. Review Comments to the Author

Please use the space provided to explain your answers to the questions above. You may also include additional comments for the author, including concerns about dual publication, research ethics, or publication ethics. (Please upload your review as an attachment if it exceeds 20,000 characters)

Reviewer #1: The manuscript provides a valuable qualitative exploration of how maternal morbidities impact women’s quality of life (QoL) across five LMICs, offering important insights into physical, emotional, social, and economic challenges during pregnancy and postpartum. The study’s strengths include its multi-country design, robust methodology, and clear thematic presentation of findings. However, there are opportunities to enhance clarity, depth, and practical relevance.

Clarity & Structure: The title and abstract could be more concise, and the introduction would benefit from a stronger justification for the focus on QoL and country selection.

**RESPONSE: We have edited the title to: ‘How maternal morbidities impact women’s quality of life during pregnancy and postpartum in sub-Saharan Africa and South Asia: A qualitative study’ and have also updated the abstract to be more concise. We now include a stronger justification for the focus on QoL in the Introduction (see lines 137-139) and a clearer description of country selection in the methods section (lines 150-151).**

Methodological Rigor: While the qualitative approach is well-detailed, further discussion on sampling limitations, socioeconomic diversity, and translation validation would strengthen transparency.

**RESPONSE: Additional details on the sampling limitations and translation process have been added to the methods section. Please see lines 173-175, 185-186, and 220-222. However, given the diversity of urbanity, access to education, and housing in each study country context, it is difficult to compare socioeconomic status across sites.**

Results & Discussion: Adding quantitative descriptors (e.g., prevalence of specific morbidities) would complement qualitative findings.

**RESPONSE: We respectfully disagree with the Reviewer about the utility of adding percentages. Given that this is a qualitative study that intends to understand women’s lived experiences, adding percentages may oversimplify and potentially distort these findings. However, we are in the process of developing a quantitative survey to assess maternal morbidities in the PRISMA study, the findings of which will be published separately.**

Regional comparisons (e.g., differences between African and Asian contexts) could be highlighted more explicitly.

**RESPONSE: While most themes and subthemes were cross-cutting, differences in some subthemes are noted in the Tables, and we now further describe differences observed across contexts for a small number of sub-themes in the text as well. Please see lines 334-335, 348-350, 367-369, 387-388, 401-402, and 485 in the Results section and lines 507 588, and 598-621 in the Discussion section.**

The discussion would benefit from concrete policy recommendations (e.g., male engagement programs, economic support initiatives).

**RESPONSE: We have incorporated concrete policy recommendations, including interventions to involve fathers in antenatal care and cash transfer programs. Please see lines 602-621 and 636-661 in the Discussion section.**

Conclusion: A stronger call to action linking findings to scalable interventions would enhance impact.

**RESPONSE: We have edited the conclusion to make a stronger call to action.**

Overall, this is a well-conducted study with significant public health implications. Addressing these refinements will further elevate its contribution to maternal health research and practice. Please see the feedback report for detailed directives; thanks.

Reviewer #2: Review

Congratulations on crafting a well-written and critical manuscript. I enjoyed reviewing your work. This manuscript identifies the physical and emotional challenges faced, factors influencing their quality of life, and lived experiences of maternal morbidities, which is of paramount importance, particularly in LMICs in sub-Saharan Africa and Southeast Asia. Below are items for review. The items are listed under the subheadings as major and minor points for your consideration.

Major concerns
1. Some quotes reveal reports of concerning themes such as violence, psychological/verbal abuse, and societal pressures/judgment/rejection. It may be helpful to ask whether interviewers were trained to provide appropriate support or referrals to participants disclosing abuse or trauma, and whether ethics protocols covered this. If this was done please mention it in the methods section. If not, please add it to the limitations section of the discussion.

**RESPONSE: Thank you for highlighting this important feature. The researchers were rigorously trained to maintain an empathetic and supportive environment during data collection without providing counseling to respondents. If a researcher noticed signs of distress or emotional upheaval in a respondent or they indicated circumstances of abuse or violence, the interview was paused till the respondent was in better control and ready to continue. Otherwise, the interview was terminated immediately, and respondents were referred to the hospital for professional support as they were open to it. See revised text in lines 269-271.**

2. Abstract line 45: The conclusions read more as the results. For example, the conclusions listed on page 18, lines 391-393, should be adapted for the abstract. Please list the primary outcomes in more detail in the abstract section as results. This can be done by providing examples. How do the results differ or agree between African and Southeast Asian countries?

**RESPONSE: We have edited the abstract results and conclusion accordingly and now include more detail about the main findings. We also now indicate that the overarching themes were cross-cutting for both African and Southeast Asian countries in the Abstract and Conclusion.**

3. Line 61: what percentage of MMR is those listed for Southeast Asia?

**RESPONSE: South East Asia accounted for 17% of all maternal deaths. This has been added to line 108.**

4. Line 67: and for East Asia or Southeast Asia? Please keep comparisons uniform throughout the introduction section.

**RESPONSE: Both South Asia and South-East Asia are used in the introduction to refer to the specific subregion that the statistics refer to, hence the difference in terminology. For example, the WHO region for which the MMR estimate derives is South-East Asia. When we describe our own study, we refer to the countries involved (India and Pakistan) exclusively as South Asian countries.**

5. Were translators needed for the direct quotes in the manuscript's results section? Did the study participant answer in English? If not, how were the quotes translated, and how were accuracy and the intended meaning maintained? Please add these methodologies and descriptions to the materials and methods section (Line 122). Please expand what is the intended meaning of “as per standardised translation protocol”? (Line 144) Is there a reference that can be provided here? Line 151: by whom were the transcripts reviewed? Although you mention asking for clarification about translation, the manuscript lacks a direct statement about the language(s) used in the interviews and whether any issues arose during translation and back-translation. It’s worth explicitly recommending that this be stated more clearly.

**RESPONSE: All focus groups and interviewers were conducted in the local languages, which are now listed explicitly in lines 185-186. The translation process for the guides and transcripts is now detailed in lines 220-230 and 238-239, respectively.**

7. To guide the reader, please add and figures to the manuscript's main text.

**RESPONSE: Figures have now been added to the main text as requested, thank you.**

8. Please expand on the intended meaning of “probe”. The authors may consider rephrasing the sentence to clarify the intended meaning.

**RESPONSE: We have now clarified the meaning of “probe.” Please see line 223.**

9. Please provide a citation for Dedoose.
a. <https://uk.sagepub.com/en-gb/eur/qualitative-and-mixed-methods-data-analysis-using-dedoose/book258543>

**RESPONSE: We have now added a citation for Dedoose.**

10. Line 184: How does the unemployment statistic compare to the national averages? This may be a more valid point for the discussion section. Are the participants skewed towards unemployment? Are LMICS more likely to be unemployed during pregnancy? Please elaborate. Does this contribute to economic challenges listed as thematic unit 4?

**RESPONSE: Thank you for the important consideration. Each site pulled national unemployment data (listed below) and compared to study findings. Overall, our study sample employment statistics are comparable with the exception of the South India site, for which ours are slightly lower (though representative of the study catchment area). Unfortunately, national data specific to employment in pregnancy is sparse in LMICs, where fertility rates are typically high among women of reproductive age. For these reasons, we have elected not to include statistics in the manuscript text.**

- **Pakistan: Regarding the employment status, according to the UN Status of Women report 2023, 34% of rural and 12% of urban Pakistani women are employed. Considering our catchment is a peri urban site, 15% seems reasonable.**
- **Zambia: The employment estimates in Zambia in the last 3 years range between ~ 25% and 50% so that the unemployment estimate of 71% in the paper seems reasonable.**
- **North India: 86.2% women aged between 15 and 29 years and 92.9% women ranging from 15-59 years provide unpaid domestic services for household members while 45.6% between 15 and 29 years and 41% women within the age range of 15-59 years render unpaid caregiving services for household members in India. The study results are corroborated by the national data.**
- **South India: In India, the female labour force participation rate was 32.8% in India in the year 2021-22, with 36% in rural areas and 23% in urban areas. World Bank reports also put it as 28,7%. This is slightly higher than our estimates, but it is consistent with hospital data.**
- **Ghana: According to the Ghana Statistical Labour statistics bulletin, the labour force participation rate in Ghana from 2022 - 2023 was 72.9% and unemployment rate of 14.7%. There are gender gaps in unemployment with rates of 17.7% and 10.9% among females and males respectively. Urba**n **unemployment rate was 18.3% percent high compared to 9.8% in rural areas.**

11. Line 333: Please provide more references for the existing literature alluded to. Can you add comparisons to other Sub-Saharan African countries or South East Asian countries?

**RESPONSE: The Salari et al. (2023) reference is a systematic review that includes 28 countries, including multiple in Asia. We have added an additional reference for the prevalence of these pregnancy-related back pain in Malawi (reference 19).**

12. Please add a succinct paragraph in the discussion section on how these challenges can be overcome. For example, can this work be used to advocate for these women's well-being, for postnatal care, food supply, transportation, and medicines in local settings, or serve as a baseline to advocate for increased awareness and education in these communities? What are the next steps following this study?

**RESPONSE: Thank you for this suggestion. We have incorporated specific recommendations throughout the Discussion section.**

13. Line 393, please provide references where this work has consistent outcomes to those listed here.

**RESPONSE: Since references are typically not cited in the Conclusions sentence, we have edited this sentence to remove mention of prior research.**

14. Line 396: how can these educational challenges be implemented?

**RESPONSE: Father-inclusive maternity care is not widely practiced, particularly in LMICs, though benefits of male caregiver involvement are well established. We added in two important references in the discussion (line 602) that address this: one, a systematic review of fathers’ educational needs in perinatal care and two, a systematic showcasing the positive association of father-inclusive care and maternal, child, and couple outcomes. In the conclusion (lines 677-679), we more clearly linked the goal of incentivizing fathers’ attendance at maternity care visits and the importance of conducting QoL assessments to educate women and the fathers of the impact of childbearing.**

15. In line 90, the authors state, "Findings will inform development of tailored interventions to improve the QoL of childbearing women in LMICs.” Can the authors please expand on these tailored approaches in the discussion section

**RESPONSE: We have incorporated specific suggestions related to routine QoL assessments in maternity care, father-inclusive care, and cash transfer programs in the Discussion.**

16. While the manuscript identifies four overarching themes, it doesn’t explicitly compare how experiences differed or were similar between countries or regions. A more nuanced discussion of cross-country comparisons may be required, particularly between Sub-Saharan Africa and South Asia. This can be done succinctly in the results section and in a short section within the discussion section

**RESPONSE: Thank you for this suggestion. While we did not conduct a formal comparative analysis, we have now elaborated on similarities and differences in women’s experiences across the different countries in the results and discussion section.**

Minor concerns
1. Line 58 “Despite recent global advancements,” please name the recent advancements succinctly.

**RESPONSE: We now explicitly mention the advancements. Please see lines 103-106.**

2. Line 62 and 63. Please add a comma after tip of the iceberg and after severe maternal morbidity

**RESPONSE: Edited accordingly, thank you.**

3. Please add a “the” before development

**RESPONSE: Edited accordingly, thank you.**

4. Replace global push with global drive

**RESPONSE: Edited accordingly, thank you.**

5. The use of AI-derived text often includes the overuse of an em dash (—). This is great to emphasise text, but a good writing tip is to limit it to once per manuscript. Overuse is distracting to the reader. For example, it is used in lines 62, 77, 78, 358 (x2), 370, 371, 388 and 388. Another tip to the authors is to avoid using “underscore” as this is widely used in AI-generated text and can become distracting.

**RESPONSE: We have removed the use of the em dash in several places throughout the manuscript.**

6. In the first sentence of the results section, please remove the word “who”.

**RESPONSE: Updated from “4 FGDs per site except Kenya who conducted 3 FGDs” to “4 FGDs per site except Kenya, where we conducted 3 FGDs”. Thank you.**

7. Please replace a with “an” FGD “participated in a FGD during pregnancy”.

**RESPONSE: Edited accordingly, thank you.**

8. In the following sentence, please change morbidities to morbidity: “In some cases, this also manifested as physical morbidities”.

**RESPONSE: Edited accordingly, thank you.**

9. Line 344 change “review on” to review “of”.

**RESPONSE: Edited accordingly, thank you.**

10. Please change increase to increasing (Line 375)

**RESPONSE: We respectfully disagree with this suggestion and have kept the word “increase” as originally written.**

11. Ensure the consistent use of a space or the percentage sign (such as “75%” vs “75 %”).

**RESPONSE: Edited accordingly, thank you.**

12. The abstract does not mention the number of study sites or countries. Pleaser add it.

**RESPONSE: We have added mention of the number of sites/countries in the abstract.**

Reviewer #3: The manuscript is well written, reads well. Just minor revision needed in the abstract.

Kindly have Introduction of the abstract to give some form of background to the reader. The aim must be concise and straightforward. The first line of the "aim section" belongs in the introduction of the Abstract.

**RESPONSE: We have updated the abstract to remove the distinct headings (i.e. Background, Methods, Results, Conclusions) per journal requirements and clarified the background component.**

6. PLOS authors have the option to publish the peer review history of their article ([what does this mean?](https://track.editorialmanager.com/CL0/https:%2F%2Fjournals.plos.org%2Fglobalpublichealth%2Fs%2Feditorial-and-peer-review-process%23loc-peer-review-history/1/010f0196c7d0e728-d9efb81e-d2d9-44d3-b4e8-b9ffc3e8a644-000000/0tH_tyAD1-SJmfxzVJdx9KrNZI05Bgywwk29iRQDGqI=210)). If published, this will include your full peer review and any attached files.


Do you want your identity to be public for this peer review? If you choose “no”, your identity will remain anonymous but your review may still be made public.


For information about this choice, including consent withdrawal, please see our [Privacy Policy](https://track.editorialmanager.com/CL0/https:%2F%2Fwww.plos.org%2Fprivacy-policy/1/010f0196c7d0e728-d9efb81e-d2d9-44d3-b4e8-b9ffc3e8a644-000000/0wvTR0yKy_gJkWy7xG85Y_8iaUKQ636iALeYjAQ8Ygw=210).

Reviewer #1: **Yes:** Ikekhwa Albert Ikhile

Reviewer #2: No

Reviewer #3: No
